# Transposable Elements, Inflammation, and Neurological Disease

**DOI:** 10.3389/fneur.2019.00894

**Published:** 2019-08-20

**Authors:** Aurian Saleh, Angela Macia, Alysson R. Muotri

**Affiliations:** Department of Pediatrics, Rady Children's Hospital San Diego, University of California, San Diego, San Diego, CA, United States

**Keywords:** LINE-1, HERV, retrotransposition, CNS, inflammation, reverse transcriptase inhibitors

## Abstract

Transposable Elements (TE) are mobile DNA elements that can replicate and insert themselves into different locations within the host genome. Their propensity to self-propagate has a myriad of consequences and yet their biological significance is not well-understood. Indeed, retrotransposons have evaded evolutionary attempts at repression and may contribute to somatic mosaicism. Retrotransposons are emerging as potent regulatory elements within the human genome. In the diseased state, there is mounting evidence that endogenous retroelements play a role in etiopathogenesis of inflammatory diseases, with a disposition for both autoimmune and neurological disorders. We postulate that active mobile genetic elements contribute more to human disease pathogenesis than previously thought.

## Introduction

Discovered in the context of maize kernel mosaicism (1), transposons are present in virtually all eukaryotes and mobilize from one chromosomal loci to another through either a DNA or RNA intermediate. They parallel viruses in many ways—with regards to their structure and function as they ensure their own survival by way of reintegration (2). Human Endogenous Retroviruses (HERV) and Long-Interspersed Nuclear Element-1 (LINE-1) are two main classes of retrotransposons and are mobilized through a “copy and paste” mechanism. HERV and LINE-1 insertions have accumulated throughout evolution and host genomes have simultaneously coevolved with these mobile elements by employing a variety of factors to suppress aberrant activity (3). LINE-1 somatic retrotransposition has been well-demonstrated to occur in neuronal lineage, however the significance of retroelement activity to normal brain function remains uncertain. Furthermore, the contribution of these elements to the symptomatology of neurodegenerative diseases is a topic of recent exploration. We begin this review with a brief overview of the types of transposable elements and their methods of integration. We will then discuss the consequences of retrotransposon activity and their dynamic relationship with various regulators. Finally, we will review the direct influence of these elements on CNS function and their contribution to disease and neuroinflammation.

## Transposable Elements: An Overview

**Figure 1:**
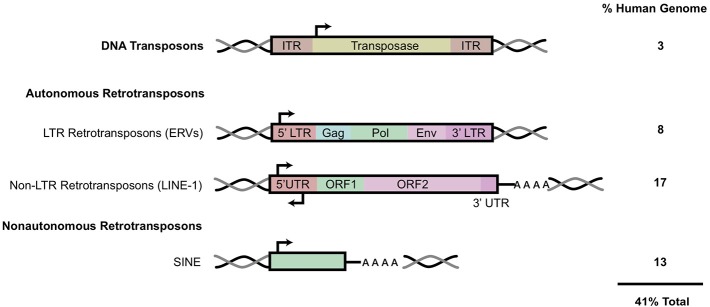
Transposable elements in the human genome. A schematic of the general structure of different classes of mobile elements. On the right is the estimated percentage present within the human genome. ITR, inverted terminal repeat; LTR, long terminal repeat; UTR, untranslated region. HERV genes include *gag*, which encodes viral structural proteins, *pol*, which encodes viral enzymes, and *env*, which encodes for the viral envelope protein. Black arrows indicate sense and antisense promoter regions. Black and gray double helices represent DNA. AAAA, represents polyA tail. This figure has been adapted from several reviews (5, 6).

**Figure 2:**
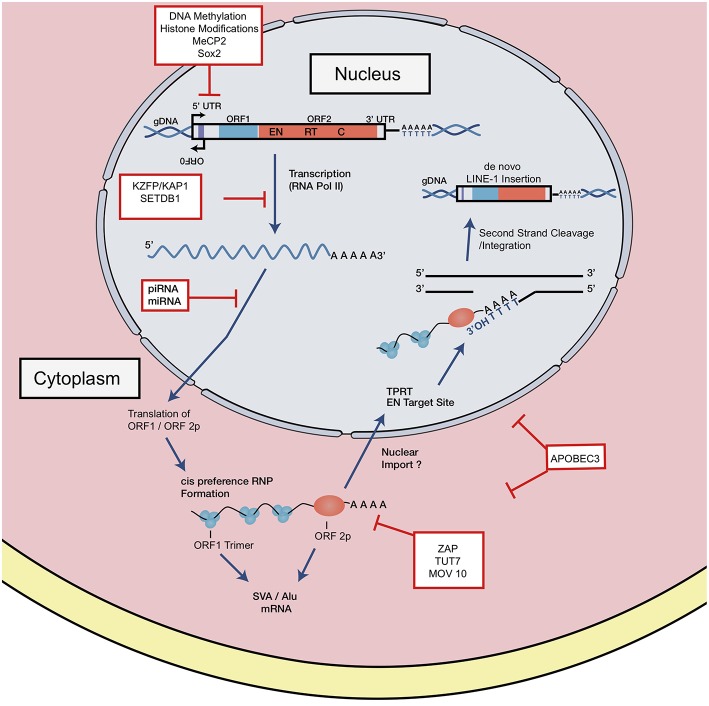
LINE-1 retrotransposition cycle and host factor regulation. Structure of full-length genomic LINE-1. 5′ UTR contains sense and antisense promoter activity. Relative positions of ORF0, ORF1, and ORF2, 3′ UTR and a poly A tail are shown. EN denotes endonuclease, RT denotes reverse transcriptase, and C denotes cysteine-rich domain. RNA polymerase II mediates transcription of retrotransposition-competent LINE-1 sequence. This transcript is exported from the nucleus where it forms an RNP complex with ORF1p and ORF2p. Through a mechanism not well-understood, the RNP is imported into the nucleus to begin reverse transcription and integration through TPRT. The ORF2 EN nicks the bottom strand of the DNA, exposing a 3′ OH, which serves as a primer for the RT to generate the cDNA from the LINE-1 mRNA. How the second strand is synthesized and integrated is a poorly understood mechanism. LINE-1 is regulated at distinct intermediates of retrotransposition, indicated in red boxes.

Transposable elements comprise at least 45% of the human genome while coding sequences occupy <3% (4). These highly repetitive strands of “junk” DNA are capable of generating new copies in the human germline and certain somatic tissues. Transposable elements (TE) can be classified as either DNA transposons or retro (RNA) transposons. The mobilization of these elements is referred to as either transposition or retrotransposition. DNA transposons, known as Class I transposons, are flanked by terminal inverted repeats and transpose with a “cut and paste” mechanism whereby the sequence is excised from one region, catalyzed by a transposase enzyme, and integrated into a separate region in the genome [Figure 1; (2, 7)]. DNA transposons constitute 3% of the genome and are no longer active in most mammals (4, 8).

Retrotransposons, also referred to as Class I transposable elements, integrate into the genome via an RNA intermediate, utilizing a “copy and paste” mechanism; this allows the active retroelements to retain their original location in the genome while accumulating copy numbers elsewhere. These retroelements can be further classified based on the presence of long terminal repeats (LTR) in the sequence. Elements such as Mammalian apparent LTR-retrotransposons (MaLR) and human endogenous retroviruses (HERV) both contain LTR sequences that flank internal coding regions (9). LTR retrotransposons comprise about 8% of the human genome [Figure 1; (10)].

Non-LTR retrotransposons can be further classified into two subtypes: LINE (Long Interspersed Nuclear Elements) and SINE (Short Interspersed Nuclear Elements) (Figure 1). Together, LINE and SINE comprise ~33% of the human genome (4). SINEs, including *Alu* and SVA elements, are non-autonomous sequences transcribed by RNA Polymerase III (11). *Alu* sequences are present in over one million copies in the human genome while SINE-R/VNTR/Alu (SVA) elements together constitute more than 10% of the human genome (4). The LINE-1 encoded proteins (ORF1/2p) recognize and bind to non-autonomous SINE sequences *in trans* to mediate their mobilization.

### HERV

HERV copies comprise 5–8% of the human genome, with some lower estimates at 1% (12, 13). HERV elements possess similar genomic organization to that of exogenous retroviruses, such as HIV. Briefly, HERV elements include gag, pol, and env regions that are flanked by LTR sequences on either side (Figure 1). The gag and pol genes encode a retroviral capsid protein and enzymes (protease, reverse transcriptase and integrase) required for viral replication and integration, respectively (12). HERVs also contain the presence of a gene encoding an envelope protein (Env), a remnant of their exogenous retroviral origin prior to their insertion and endogenization into germline cells (14). Functional env proteins have been shown to initiate innate and adaptive immune responses (15).

Transcriptionally active HERV subfamilies have more recently been implicated as pathophysiological contributors to various disorders (Table 1). An initial 1993 study reported that the addition of herpes simplex virus type 1 (HSV-1) to primary leptomeningeal cells isolated from a patient with multiple sclerosis (MS) lead to robust co-expression of retroviral-like particles and reverse transcriptase activity (16). The original multiple sclerosis associated retrovirus (MSRV) sequence identified in the virus-like particles was shown to be highly represented in human DNA but never characterized until MSRV-specific primers defined the previously unknown HERV-W subfamily (17, 18).

**Table 1:** Disease and associated retroelements.

**Disease**	**Retrotransposon**	**Mutation/Gene Referenced**	**Elevated Cytokines**	**CNS vs. Systemic**
Multiple Sclerosis (MS)	HERV-W		IFNγ, IL-6, TNF-α	CNS
Aicardi-Goutieres Syndrome (AGS)	LINE-1	TREX1, RNaseH2	TNF-α, IL-15, IFN-α	CNS
Rett Syndrome (RTT)	LINE-1	MeCP2	IL-6, IL-8	CNS
Sporadic Amyotrophic Lateral Sclerosis (ALS)	HERV-K	TDP-43	TNF-α, IL-6, IL-8, IL-1β	CNS
System Lupus Erythematosus (SLE)	HERV-E	Sgp3	IL-15, IL-10, IFN α/β, IL-6	Systemic
Aging-related pathologies	LINE-1	SIRT6	IFN	Systemic
Autism Spectrum Disorder (ASD)	LINE-1		IFNγ, IL-1β, IL-6	CNS

*Diseases listed with associated endogenous retroelement contributions and observed cytokine expression in vivo or in vitro. Genes listed were ones referenced in the main text. Last column addresses whether effects are primarily systemic or localized to CNS*.

This work provided the initial evidence for retroviral-triggered HERV activation as a contributor to the pathology in neurological disorders (16). Similar reports have shown that addition of other exogenous retroviruses, such as HIV-1 and HTLV-1, result in increased expression of HERV-W and HERV-K Env proteins, resulting in a mis-regulated immune response (19). Implications of these elements in neurological diseases sporadic amyotrophic lateral sclerosis (ALS) and multiple sclerosis (MS) will be discussed more extensively later in the review (Table 1).

### LINE-1 Retrotransposons

LINE-1 elements are ubiquitous, autonomous retrotransposons with an estimated 500,000 copies contained within the human genome (3, 4). A majority of LINE-1 copies are immobile and unable to retrotranspose, due either to 5′ truncations or inversions introduced into the sequence (20–23). Approximately 80–100 copies are mobile (labeled retrotransposition-competent LINE-1 or RC-L1) (24). Of these, six highly active, or “hot” LINE-1s are responsible for a bulk (84%) of the retrotransposition activity, according to an in-culture cell retrotransposition assay (24).

Full length, RC LINE-1 are 6 kb in length and contain a 5′ untranslated region (5′ UTR), two open reading frames (ORF1, 2), and a 3′ UTR punctuated with a poly-A tract (25) (Figures 1, 2). The LINE-1 promoter region has no TATA-box and displays both sense and antisense activity within the 5′ UTR (25, 26). Additionally, a primate-specific antisense ORF0 of unknown function has also been described within this region (27). The LINE- 1 ORF1 encodes for a protein that has nucleic acid chaperone activities and an RNA binding domain (28, 29). ORF2 encodes for a protein with enzymatic activity strictly required for LINE-1 retrotransposition. ORF2p has both endonuclease (EN) and reverse transcriptase (RT) activities; equally critical for target site cleavage and integration [Figure 2; (30, 31)].

The LINE-1 transcription start site begins at the RNA polymerase II promoter region, or more precisely, within the first 100 base pairs of the 5′ UTR (25, 32). Once transcribed, the full-length capped and polyadenylated LINE-1 mRNA is exported into the cytoplasm where it combines *in cis* with ORF1 and ORF2 proteins to form a ribonucleoparticle (RNP) complex (Figure 2) reviewed in Doucet et al. (33), Macia et al. (5), and Elbarbary et al. (34). In a more traditional model, the RNA-protein complex can be imported back into the nucleus by a mechanism not well-understood, where target-primed reverse transcription (TPRT) might take place. The RNP complex binds preferentially to an AT rich consensus target (5′ TTTT/AA 3′ and variants) recognized by the EN (35–37). The EN nicks the bottom DNA strand, exposing a 3′ OH which primes the reverse transcription of the LINE-1 mRNA template into cDNA. Recent observations have challenged this model, suggesting that TPRT might also start in the cytoplasm or either unfinished retrotransposition events might be transported back to the cytoplasm (38). The complete mechanism of second DNA strand cleavage and subsequent cDNA synthesis is unknown, but recent studies have suggested that RNase H2 is required to degrade the RNA:cDNA hybrid generated during this step (39).

### Heterogeneity of Insertional Preferences and Distribution of Retrotransposons Across Different Populations

Selective processes and insertion bias impact the distribution of LINE-1 elements in the genome (40). The distribution of retrotransposons within the genome is variable; SINEs are more aptly tolerated by the cell and have been shown to localize in gene-rich (GC-rich) regions whereas pre-existing, static LINEs are highly enriched in intergenic (AT-rich) isochores, likely due to the length of their insertion sequences (34, 41).

A recent meta-analysis of engineered LINE-1 insertions by Flasch and colleagues reported a strong bias for LINE-1 insertional preferences. Wild-type LINE-1 EN preferentially nicks the lagging strand of DNA replication fork, resulting in cDNA insertions into leading strand templates (42). It was determined that varied levels of open chromatin state had only minor influence on LINE-1 insertion preference. A similar study recently published by Sultana and colleagues corroborated these findings. *De novo* LINE-1 retrotransposition was induced in cultured HeLa S3 cells followed by ATLAS-seq profiling to detect and map integration sites. This study also confirmed minimal association (2–3%) of all insertions in chromatin segments annotated as weak enhancers or with histone modifications characteristic of weak enhancers (H3K4me1) (43). The strongest association was enrichment of LINE-1 insertions within early replicating regions of the genome. Insertion orientation was shown to be influenced by the directionality of the replication machinery; EN cleavage of the bottom strand was highly enriched when replication fork moves leftward. Both studies provide evidence that LINE-1 integration events do not target expressed genes, open chromatin or transcribed regions but instead associate with host DNA replication (42, 43). These findings provide the first clues that LINE-1 integration and DNA replication may be mechanistically linked.

**Figure 3:**
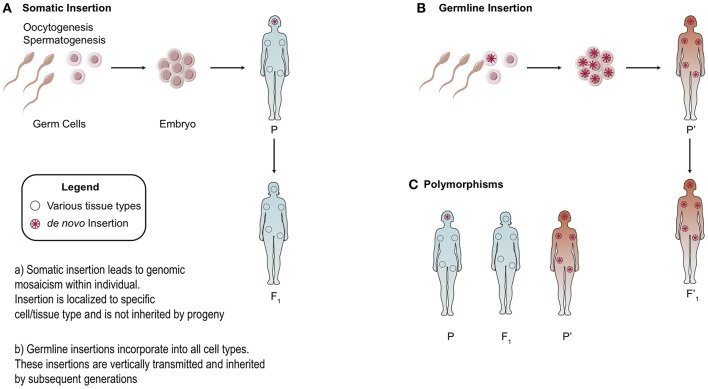
Somatic vs. Germline Insertions. Differences between the heritability of germline vs. somatic retrotransposon insertions. **(A)** Somatic insertions lead to mosaicism within the individual but are not inherited. **(B)** Generally, germline or early embryonic insertions are incorporated into the three germ layers and are present in all tissue types. **(C)** Example of polymorphisms across different populations based on the presence or absence of an insertion.

There is heterogeneity in the distribution of endogenous retroelements across different human populations: from the presence/absence of the element to single nucleotide polymorphisms (SNPs) [Figure 3; (20, 44, 45)]. Because RC-L1 mobilize to novel insertion sites, it is logical that individuals will carry differences in the presence or absence of LINE-1 insertions at various loci in their genome. Polymorphisms in retroelement insertions are generated either in the germline or early in embryonic development (Figure 3). Germline insertions are incorporated into all tissue types within the individual and are heritable by the next generation. Somatic *de novo* LINE-1 insertions, which occur later in development, are not inherited by subsequent generations and are localized to the cell(s) in which the insertion occurred (Figure 3).

As a consequence, polymorphisms can generate variability in activity levels of retrotransposons. Indeed, allelic variability within the LINE-1 elements was demonstrated to have up to 16-fold differences in activity (44). To study the differential expression of polymorphic LINE-1 elements, Philippe et al. mapped active LINE-1 HS-Ta (human-specific) copies according to their epigenetic signatures in 12 somatic cell lines. A restricted subset of polymorphic LINE-1 loci remain highly active but are differentially regulated according to cell type (46). ORF1p expression was high in neural progenitors and cancer cell types studied while little to no expression was seen in primary fibroblasts, consistent with previous work (47–49). In summary, LINE-1 transcription in somatic cells was shown to be governed by locus- and cell-type- specific determinants (46).

Polymorphisms are observed with HERV elements as well. Next generation sequencing (NGS) characterized unfixed HERV-K insertions across different human populations (14, 50). Scans for polymorphisms in HERV-K loci revealed 17 loci in the individuals studied that were not present in the human reference genome. On average, each individual possessed six loci, often in the heterozygous state, that were not found in the reference genome (50). A separate study analyzed 36 non-reference polymorphic HERV-K proviruses from more than 2,500 globally sampled individuals found insertion frequencies ranging from <0.0005 to >0.75, and varied by population (14).

In summary, a myriad of selective processes influences the distribution and insertional preferences of retrotransposons. This allows for genomic mosaicism within a single individual as well as polymorphisms across different human populations.

## Cellular Impact of LINE-1 Retrotransposition

**Figure 4:**
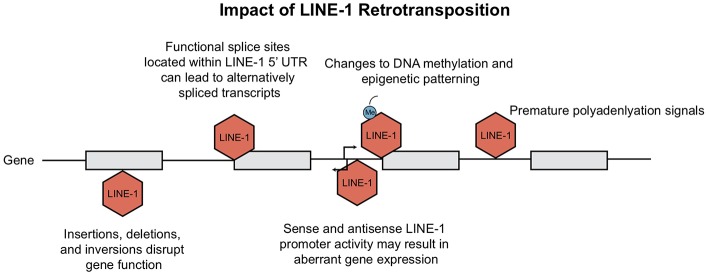
Consequences of LINE-1 retrotransposition. Ongoing retrotransposition generates genomic instability and consequently interferes with host gene expression at many levels. Consequences include disruption of gene function due to LINE-1 insertions, as well as generation of mis-spliced or prematurely truncated transcripts. Insertions have been shown to impact host gene expression through changes in epigenetic patterning and LINE-1 promoter activity. This figure is adapted from Macia et al. (5) and Garcia-Perez et al. (6).

There are innumerable ways in which retrotransposons can influence the genome and impact cellular function (Figure 4). LINE-1 elements generate structural variation/instability within the genome and subsequently interfere with host gene expression. In this section, we review the insertional and transcriptional impact of LINE-1 elements in the human genome.

The current rate of LINE-1 retrotransposition has been estimated to occur between 1 out of 20 and 1 out of 200 births, depending upon the method used in the analysis (10). LINE-1 insertional mutagenesis has resulted in over 120 cases of spontaneous or inherited disease; including diseases such as hemophilia A, cystic fibrosis, and breast cancer; reviewed in Chen et al. (51). Apart from mutations caused by insertions, DNA recombination of chimeric LINE-1 can lead to retrotransposition-mediated deletions, duplications, or rearrangements (52, 53).

Insertional mutagenesis is not the only hazard for our cells, the presence of both sense and antisense promoter within the 5′UTR of LINE-1 elements can activate upstream or downstream transcription [(34, 54, 55); Figure 4]. Among all the transcriptionally active elements present in the human genome, the strength of LINE-1 promoters has been shown to be sequence and context-dependent. Lavie et al. generated 5′ UTR constructs to test observable differences in promoter strength. Although there was no clear link between nucleotide variations and transcriptional activity, deletion of 5′ genomic flanking sequences from the constructs resulted in both enhanced and diminished promoter activity, depending on whether the sequence acted as an enhancer or repressor (32). As for the LINE-1 antisense promoter, Matlik and colleagues established its activity as tissue specific; driving transcription of adjacent genes to yield chimeric transcripts of host genes [Figure 4; (56, 57)].

Not only does the activity from the LINE-1 promoter at insertion sites contribute to aberrant host gene expression; LINE-1 insertions can also create splicing variants, generate mis-spliced or prematurely truncated transcripts, promote transcriptional termination or even promote changes to our epigenome (Figure 4). Splicing of pre-mRNAs is a tightly regulated process as it contributes to proteomic diversity and modulates gene expression. RNA extracted from human Ntera2 and Sk-Br-3 cancer cells, which express high levels of LINE-1 transcripts, identified many functional splice sites within the 5′UTR (58). It was demonstrated that several of these LINE-1 splice variants are capable of undergoing retrotransposition. Upon insertion, these active splice sites can disrupt normal gene expression via alternative splicing of mRNA transcripts (58). Similarly, *Alu* elements harbor consensus sequences that resemble 5′ and 3′ splice site signals, contributing to modifications in pre-mRNA splicing (59, 60). In addition, the presence of LINE-1 adenosine-rich insertions can give rise to new polyadenylation signals, resulting in transcriptional termination [Figure 4; (61)].

LINE-1 elements not only create changes in splicing; LINE-1 loci that contain premature stop codons may still encode for truncated ORF2 proteins with a retained functional EN domain (62). It was suggested that EN activity of ORF2p may generate nicks independent of retrotransposition and could therefore contribute to double stranded breaks (DSBs) formation (63). In fact, the number of observed LINE-1 induced DSBs was demonstrated to be greater than the predicted numbers of successful insertions (63).

Finally, LINE-1 can also alter the epigenome; methylation of LINE and SINE CpG islands leads to “epigenetic patterning” and subsequent silencing of neighboring gene promoters [(64); Figure 4]. Baylin et al. demonstrated that hypermethylation stemming from human *Alu* elements can silence tumor suppressor genes (65) which has large implications for their role in cancer. Alternatively, *hypo*methylation of the LINE-1 promoter was shown to directly activate an alternate transcript of the MET oncogene in bladder tumors; inducing ectopic gene expression and possibly altering disease susceptibility (66). In summary, new TE insertions have been shown to have many profound effects on the genome. A contributor to genomic variation, retrotransposons alter host gene expression at the epigenetic, transcriptional and translational level. Host cells have simultaneously evolved many responses in order to repress TE activity, which we review in the next section.

## Cellular Responses to Retrotransposition

Intrinsic immune responses to viral pathogens are co-opted to restrict retroviruses and retrotransposons as well. Given that these retroelements can impact the cell in a myriad of ways, the host genome has coevolved to employ a variety of responses to repress aberrant activity.

### LINE-1 Transcriptional Repression

Transcriptional repression is a major mechanism of TE regulation and can be achieved with the deposition of repressive epigenetic modifications. DNA methylation, in the form of 5-methylcytosine (5 mC) and N6-methyladenine (6 mA) are widely used chemical modifications in eukaryotes and higher organisms [Figure 2; (67)]. Waves of *hypo*methylation during embryogenesis are linked with higher rates of retrotransposition. This effect has been replicated in embryonic stem cells (ESC) and induced pluripotent stem cells (iPSCs) (68–70). In preimplantation mouse embryos, which are exceptionally hypomethylated, genomic integrity is maintained with factors such as histone chaperone chromatin assembly factor 1 (CAF-1) (71). CAF−1 was shown to mediate the replacement of Histone variant 3.3 (H3.3) with H3.1/3.2, which serves as a repressive histone modification and subsequently protects the embryo from retrotransposon activity [Figure 2; (71)]. When histone variant H3.3 was deleted from embryonic stem cells, trimethylation of histone 3 on lysine 9 (H3K9me3) was shown to be reduced at HERV sites, establishing an important link between H3.3 and endogenous retrovirus silencing (72).

Transcriptional silencing of retrotransposons can be induced by DNA methylation or histone modifications, but TEs also harbor binding sites for many transcription factors, enabling context-specific transcriptional regulation. The Kruppel-associated box (KRAB)-containing zinc finger proteins (KZFPs) are key regulators of TE activity—often repressing TEs expressed in early embryos (73, 74). Mechanistically, repression is mediated once the C-terminus tandem array of zinc finger motifs binds to target TE sequences and the KRAB domain recruits and tethers to the cofactor KAP-1 (KRAB associated protein 1). KAP-1 then functions as a scaffold for chromatin modifying complexes such as SETDB1 (Set Domain Bifurcated-1) and H3K9 methyl transferase [Figure 2; (75, 76)].

In order for KAP1 to target a specific retroelement, the associating KRAB-ZFP must evolve to bind to that specific regulatory sequence. This is usually followed by mutations in this sequence, such that retrotransposons evade repression; a true evolutionary arms race (77). A recent study by Trono et al. elucidated the role of KZFPs on TEs during embryonic genome activation (EGA), demonstrating that a large proportion of TE-embedded regulatory sequences have been co-opted to serve as lineage- or tissue-specific enhancers of gene expression (78). Authors observed clustered TE sequences of the evolutionarily recent SVA, LTR5Hs-HERVK, and LTR7-HERVH in human embryonic stem cells (hESC) during EGA. These sequences were shown to strongly associate with members of the Kruppel-like factor (KLF) family of transcription factors, notably KLF4. Clustered regularly spaced short palindrome repeats—interference (CRISPRi) targeting of these TE sequences led to up- and downregulation of genes near the target vicinity. For example, CRISPRi of an LTR5Hs-based enhancer resulted in a significant downregulation of PRODH, a neuron-specific gene located 2 kb downstream from the enhancer. Thus, TEs which possess embedded enhancer sequences could subsequently exert large transcriptional influence in hESCs during EGA. By examining the degree of conservation within the zinc fingerprints, evolutionarily recent human KZFPs were shown to target TE subfamilies of similar ages (78). In sum, these recent studies demonstrate the intricate, coevolutionary dynamic between KZFPs and TEs.

However, there is some evidence indicating that this arms-race hypothesis may be too simplistic (79, 80). Imbeault et al. performed a clustering and aging analysis of various KZFPs to estimate their evolutionary ages. These conserved sequences were aligned against various transposable element subfamilies; many transposable element-KZFP pairs were found to be highly conserved long after the transposable elements lost their ability to mobilize (80). In some cases, KZFPs appear much earlier than their TE target, illustrating a complex co-option model where specific regulatory networks are established (78, 80). This is an example whereby the host facilitates the impact TEs can have by regulating their disruptive capacity. In addition, host cells even use conserved TE sequences to their advantage by setting up tissue-specific transcriptional networks.

### Post-transcriptional Regulation of LINE-1 Elements

LINE-1 can be regulated post-transcriptionally by small RNAs, like microRNAs (miRNAs) or PIWI-interacting RNAs (piRNAs). Small RNAs can act via targeted RNA degradation, reviewed in Heras et al. (81) and Mita and Boeke (82). The role of RNA interference (RNAi) effectors in regulating TE transcripts is substantial; RNA-induced silencing complex (RISC) pathways are common cellular processes that utilize endonucleolytic cleavage to degrade TE transcripts (83, 84). Mutations to the Dicer protein, a component of the RISC complex, result in elevated transcription of LINE-1 elements (3). The recent discovery of small, non-coding miRNAs also highlighted their critical regulatory role. It was recently shown that miRNA-128 restricts LINE-1 activity via two mechanisms (Figure 2). It can directly target ORF2 RNA for degradation or target the 3′ UTR sequence of required cofactor TNPO1 (nuclear import factor transportin 1) (85). TNPO1 has been proposed to facilitate transport of LINE-1 RNP complex into the nucleus by binding to nuclear localization signals (85). Another recent study identified a separate cellular target of miRNA128-mediated LINE-1 repression (86). hnRNPA1 (heterogeneous nuclear ribonucleoprotein A1) binds to poly(A) sequences in mRNA to facilitate nuclear shuttling (87, 88). hnRNPA1 has been described to interact with LINE-1 ORF1p within the RNP complex and with TNPO1, through its nuclear localization signal (89, 90). miRNA128 was shown to repress hnRNPA1 by directly binding to the coding sequence of hnRNPA1 mRNA, significantly reducing levels of *de novo* LINE-1 retrotransposition (86). piRNAs are important repressors of TE activity in the germline (Figure 2). They interact with the PIWI subfamily of Argonaute nucleases and have been shown to cleave TE transcripts in cytoplasm as well as recruit repressive histone modifiers to silence transcription (91). Intact piRNA pathway was demonstrated to be necessary for *de novo* methylation of LINE-1 transgene in male mice testes (92). If the LINE-1 transcript is not targeted for degradation, the cell will employ other host factors to target downstream complexes within the retrotransposition cycle.

### Post-translational Mediated Repression

The LINE-1 RNP complex, a retrotransposition intermediate, is commonly targeted for destabilization and degradation (93). The zinc-finger protein ZAP, in addition to targeting several viral families, has been suggested to colocalize with LINE-1 RNA in cytoplasmic stress granules to promote loss of RNP integrity and inhibit LINE and *Alu* retrotransposition [Figure 2; (94–96)]. Post-transcriptional modifications of LINE-1 mRNAs within the RNP complex offers another way of restricting mobility. TUT7 (terminal uridyl transferase 7) in cooperation with MOV10, transfers uridine residues to LINE-1 mRNA in the cytoplasm. MOV10, a helicase, displaces ORF1p to allow for cytoplasmic 3′ uridylation—ultimately inhibiting ORF2p RT initiation within the nucleus [Figure 2; (97)]. In addition, the APOBEC family of enzymes, specifically APOBEC3G and APOBEC3F, have been shown to work through a process independent of cytosine deamination to selectively inhibit *Alu* retrotransposition, possibly by destabilizing the RNP complex [Figure 2; (98)]. APOBEC3B/F also strongly interfere with LINE-1 activity; catalytically inactive APOBEC mutants maintained LINE-1 inhibition, also indicating a deamination-independent mechanism (99, 100), while APOBEC3A has been proposed to localize in the nucleus to deaminate the transiently expressed LINE-1 ssDNA that appears during integration and prevent retrotransposition (101). Even with the plethora of host mechanisms put in place to repress endogenous retroelements, *de novo* insertions still take place within somatic tissues, with substantial LINE-1 retrotransposition occurring in neural lineages.

## LINE-1 in the Developing Brain

A plethora of evidence supports that both endogenous and engineered LINE-1 retrotransposition can occur pre and post-mitotically in the healthy and diseased brain, reviewed in Suarez et al. (102). New retrotransposition events can alter gene expression and ultimately influence cellular phenotype; in the healthy brain this is thought to contribute to neuronal somatic diversification (49, 103). Through the use of an engineered LINE-1 element tagged to a retrotransposition-indicator cassette, retrotransposition events were shown to occur *in vitro* with adult rat neural progenitor cells (NPCs) and in the brains of mice *in vivo* (103). This was one of the earliest documented cases of somatic retrotransposition occurrence *in vivo* (103). A few years later, endogenous LINE-1 mRNA was shown to be detectable in NPCs isolated from human fetal brain cells (47). To investigate endogenous LINE-1 activity, a copy number variant (CNV)-based qPCR assay was performed on genomic DNA extracted from various tissue types of healthy human adults. Interestingly, it was reported that ORF2 content in the hippocampus was consistently higher when compared to heart or liver samples from the same individual (47). Few years later, by using Retrotransposition-capture sequencing (RC-seq), a high throughput sequencing method that targets LINE-1 5′ and 3′ ends, applied to DNA extracted from various tissues again identified significantly higher ORF2 copies and LINE-1 CNV within the hippocampus (49). Separate studies implemented single-cell RC-seq on hippocampal neurons and resulted in an estimated 1.2–13.7 somatic LINE-1 insertions per neuron (104–106), although the frequency of *de novo* insertions per cell is highly debated (107). The hippocampus, being one of the few brain regions consisting of a neurogenic niche, is ubiquitous with genomic LINE-1 mosaicism. Because LINE-1 retrotransposition events have increased occurrence during neurogenesis (103, 108), it is likely that LINE-1 activity rises in a region like the hippocampus (47). Interestingly, in a recent analysis of 24 hippocampal neurons using RC-seq, whole genome sequencing (WGS) and LINE-1 insertion profiling revealed that somatic insertions which occur during neurodifferentiation in hESCs may occur due to mutations at the Ying Yang 1 (YY1) transcription factor binding site (109). This YYI binding site in the LINE-1 promoter region is known to mediate CpG island methylation and epigenetic repression. Specific loci without an intact YY1 binding site were shown to generate cortical and hippocampal neuron LINE-1 insertions, corroborating an underlying epigenetic mechanism for LINE-1 retrotransposition during neural development (109).

Retrotransposition during neural development may contribute to “genome plasticity” and neuronal diversity by allowing for variation in genomic DNA from cell to cell. By studying the effects of retroelements during neurogenesis, one can examine the early fate choices between different cell lineages (103). Muotri et al. reports a 10-fold increase in LINE-1 promoter during the first 24 h of neuronal induction, consistent with downregulation of the Sox2 promoter (103). It was proposed that subtle changes to LINE-1 promoter methylation may explain the selective activity levels in NPCs; perhaps that the LINE-1 promoter is temporarily released from epigenetic suppression during neurogenesis (47, 49). Indeed, temporal methylation patterning of the LINE-1 promoter during neurogenesis was analyzed in a fairly recent study; researchers applied RC-seq to observe distinct DNA methylation profiles for *de novo* LINE-1 insertions in an hiPSC line (108). RC-seq performed on cells throughout various timepoints of fibroblast reprogramming and neurodifferentiation identified two well-characterized *de novo* LINE-1 insertions. General trends showed, on average, ~60% methylated CpG dinucleotides in fibroblasts and mature neurons, while only ~30% were methylated in iPSCs. Surprisingly, they observed a 23% reduction in methylation in day 112 neurons when compared to earlier day 72 neurons. This was followed by a significant 20% increase in methylation at day 156 neurons (108). This study establishes a dynamic temporal patterning of methylation for the LINE-1 promoter during neurodifferentiation. The evidence collected on LINE-1 mobilization in both the developing and adult brain, opens new questions about their contribution to somatic mosaicism, aging and neurological diseases.

## Mobile Elements and Neurodegenerative Disorders

There has been an increasing interest in studying endogenous retroelements as their activation has been observed and implicated in a variety of neurological disorders (Table 1). For example, all three HERV-K structural genes (gag, pol, env) have been shown to have increased expression in patients with sporadic ALS when compared to healthy controls (110). ALS is neurodegenerative disease characterized by loss of both upper and lower motor neurons. A number of studies have established the presence of retroviral RT activity in the serum of ALS patients (111, 112). Higher expression of HERV K-Env—a powerful immunopathogenic envelope protein—is observed in cortical pyramidal and spinal neurons in post-mortem brain tissue of ALS patients (110). It is not currently known what triggers expression of HERV-K in adult neurons; however, activation of HERV-K genes was shown to decrease dendritic length, branching, and complexity of transgenic mice motor neurons (110).

Abnormalities in transactive response DNA-binding protein 43 (TDP-43) is likewise observed in the majority of sporadic ALS cases (Table 1). TDP-43 is a dimeric nuclear protein and part of the heterogenous nuclear ribonucleoprotein family (hnRNP) (113). In the CNS, its function is broadly categorized as a regulator of pre and post-transcriptional events as it binds to UG-rich motifs in single-stranded RNA/DNA (113, 114). Crosslinking immunoprecipitation (CLIP-seq) and chromatin immunoprecipitation (ChIP) data exhibits TDP-43 binding broadly to retrotransposon-derived transcripts in human brain tissue and directly to the HERV-K LTR sequence, respectively (110, 115). A study conducted by Lisa Krug et al. addressed whether functionally abnormal TDP-43 expression in *Drosophila* causes a derepression of retrotransposable elements and if so, whether this contributes to a degenerative phenotype (116). Expression of human TDP-43 (hTDP-43) was shown to induce broad retrotransposon transcript expression in *Drosophila* neurons and glia. Glial expression of hTDP-43 causes a remarkable reduction of Dicer-2/Argonaute2 mediated silencing while causally inducing DNA-damage mediated cell death (116). In a more recent study, analysis of diseased neuronal nuclei from brain tissue of patients with frontotemporal degeneration ALS (FTD-ALS), provides insight for molecular changes associated with TDP-43 loss. Liu et al. utilized fluorescence-activated cell sorting (FACS) to fractionate diseased neurons followed by ATAC-seq to quantify chromatin accessibility (117). Loss of nuclear TDP-43 was associated with chromatin decondensation around LINE-1 elements. Although it is unclear whether decondensation of LINE-1 elements is a direct result of TDP-43 protein loss, there appeared to be some specificity or preference in decondensation of LINE-1 elements over other repetitive sequences (117). Together, these findings in humans and vertebrate models, suggest that unregulated retroelement expression is somewhat involved in the pathology of ALS, although there is no evidence that it is the primary cause of the syndrome. Potential therapy in the form of antiretroviral RTi is also underway in clinical trials of ALS patients (118, 119).

Sporadic Alzheimer's Disease (SAD) has also been linked to retroelement activation; however, studies have displayed conflicting results on whether LINE-1 sequences are upregulated in patients with SAD (120, 121). “Mosaic genomic recombination events” were observed within the Alzheimer's-related gene, amyloid precursor protein (APP), in neurons of patients with SAD (122). These variants, which lacked intronic sequences, were termed “genomic cDNAs” (gencDNAs). It was hypothesized that they originated from RNA and required endogenous RT activity to insert into double strand breaks. Addition of nucleoside reverse transcriptase inhibitors (nRTi) abacavir (ABC) and azidothymidine (AZT) prevented the production of gencDNAs, further encouraging their therapeutic potential (122). In order to determine the effect of Alzheimer's Tau pathology on TE activity, wild-type and Tau mutant Drosophila were profiled for TE activation including 8 LTR retrotransposons and 4 non-LTR retrotransposons (123). Authors observed a significant increase in expression in three of the 12 TEs assessed in the mutant fly brains (*copia, gypsy* and *het-a*) suggesting a possible Tau- associated mechanism for TE activation (123).

Retroelement recombination events are also implicated in early onset Parkinson's Disease (PD). Whole genome sequencing analysis of three families with early onset PD revealed five different structural variations in the PRKN (parkin RBR E3 ubiquitin protein ligase) gene. Structural variation formation is proposed to occur with non-allelic homologous recombination. LTR and non-LTR retrotransposon sequences were identified within two kilobases of the deletion break point, suggesting that the deletions may have originated due to retrotransposition events (124). The link between retroelement activation in neurodegenerative disorders is present but not fully established. We consistently see an upregulation of retroelements in the diseased state, but we do not fully understand how this is initiated or whether it directly contributes to disease pathology. The following section will review the evidence which supports endogenous retroelements as initiators of inflammation and subsequent inflammatory responses in the diseased state.

## TEs and Inflammation

**Figure 5:**
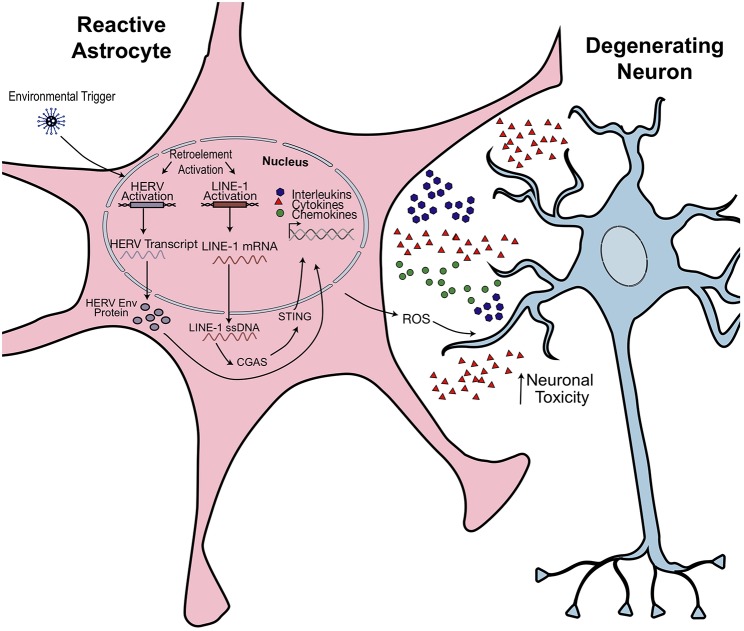
Hypothesized model of retroelement-mediated neuroinflammation. Environmental or cellular triggers may activate retrotransposon transcription. Production of pathogenic HERV Envelope proteins (Env) or cytoplasmic accumulation of LINE-1 ssDNA activates an innate immune response. Astrocytes become activated, releasing proinflammatory cytokines and reactive oxidative species (ROS). This has toxic effects on neighboring neurons and could promote morphological and synaptogenic defects, subsequently promoting neuropathophysiological effects. Figure is adapted from Thomas et al. (38).

The immune system protects against viral infections through coordinated innate and adaptive immune responses and while the contribution of innate immunity to anti-viral defenses has been extensively studied, little is known about the contribution of transposable elements to immune responses exempt of viral infection. When viral DNA is present in the cytoplasm, it triggers activation of the cGAS-STING pathway, subsequently producing interferons to initiate an inflammatory response (Figure 5). The interferon responses elicited during the targeting of virus-infected cells may be mechanistically linked to deregulating retroelement production (125). There is mounting evidence that endogenous retroelements play a large role in initiating neuroinflammation. Endogenous nucleic acid detection by the innate immune system underlies many autoimmune diseases (126) When there is an inflammatory response but no viral infection, what is the role of retroelements? More specifically, how are these engaging the pathophysiological pathways leading to features of the disease pathology?

There is increasing interest in studying endogenous retroelements as contributors to a variety of inflammatory and neurodegenerative disorders. Diseases such as MS and Aicardi-Goutières syndrome (AGS) have retrotransposon intermediates linked as key effectors of inflammation (16, 38, 127). In addition, there are now several studies that link aging, TEs, and inflammation.

### Aging

Mechanisms repressing LINE-1 activity are shown to be less efficient during the aging process. A recent study by De Cecco and colleagues demonstrates increased LINE-1 transcript levels in senescent cells. The accumulation of the cytoplasmic LINE-1 cDNA drives expression of the senescence- associated secretory phenotype (SASP). A type-I interferon (IFN) response is typical with age-associated inflammation in several tissues (Table 1). Liver and adipose tissue of 26-month-old mice showed significant increase in LINE-1 mRNA expression when compared to mice at 5 months. IFN-I and SASP response genes were assessed by RT-qPCR and showed the same trend. This response is antagonized through the use of reverse-transcriptase inhibitors (RTi). Mice treated with RTis demonstrate a reduced IFN response and associated inflammation (128). However, RTis have exhibited an intrinsic anti-inflammatory property, leading some to believe the effects as non-specific (129). The effects of LINE-1 activity in aging were also examined in the mono-ADP-ribosylase/deacetylase protein SIRT6 (Silent Mating Type Information Regulation 2 Homolog 6) deficient mice. SIRT6 KO mice display a severe aging phenotype, with a lifespan of 35 days (130). SIRT6 KO mice demonstrate high levels of LINE-1 expression due to SIRT6's repressive role in ribosylating KAP1 (131, 132). Without SIRT6, LINE-1 cytoplasmic DNA levels increased, triggering cGAS (cyclic GMP-AMP synthase) -mediated IFN response. Treatment with the RTi inhibitors lamivudine and stavudine, significantly expanded the lifespan of the mice while also improving body mass, mobility and behavior phenotypes (130). These results further implicate LINE-1 as a contributor to the pathology of age-related diseases. Although further investigation is needed regarding the mechanisms in which new L1 copies are generated in the cytoplasm, these novel data encourage the potential therapeutic use of RTis for various age-associated conditions.

### Multiple Sclerosis

Following the original identification of retroviral-like particles (16), additional research revealed increased RT activity and HERV protein production with Herpesviridae stimulation of various human cells *in vitro* (133–136). MS is an hyperinflammatory, demyelinating disease of the CNS with no known cure. The disease is characterized by lesions in white matter that lead to blood-brain barrier breakdown and axonal disruption down the spinal cord (137). In addition to genetic and environmental factors, expression of HERVs is now considered a risk factor for MS disease progression (135). The retroviral-like particles isolated from cell culture supernatants of MS patient samples, referred to as MS-associated retroviral agent (MSRV), were demonstrated to originate from HERV elements, as mentioned previously (17, 18, 138). Herpes virus is suggested to upregulate HERV-W expression, which encodes for its own immunopathogenic envelope protein (Env). *In vitro* stimulation with HERV-W Env proteins displays activation of innate immune responses through pattern recognition receptors TLR4 and CD14, leading to considerable proinflammatory cytokine production (134). Peripheral blood mononuclear cells, isolated from relapse-remitted MS patients and stimulated with the MSRV Env protein, induces elevated IFNγ, IL-6 and TNF- α expression (134, 139). Clinical trials performed on MS patients with the antiretroviral integrase inhibitor, raltegravir, failed to see reduction in lesion count, progression, or inflammatory cytokine levels, suggesting HERV W-Env protein production is not targeted with this integrase (140).

### Systemic Lupus Erythematosus

Elevated HERV transcription has been implicated in systemic lupus erythematosus (SLE) pathogenesis. HERV-E mRNA expression levels were found to be higher in lupus CD4+ T cells than in cells from healthy controls (141) but the full contribution of HERV activity to SLE etiology is not known. Deletions in the genes encoding for the KZFP, SNERV1/2 (suppressor of non-ecotropic ERV-1/2) resulted in a 2-fold increase in gene expression of six genes directly overlapping a non-ecotropic ERV sequence (NEERV) (127). The NEERV envelope glycoprotein gp70 is a major immunoantigen and promotes nephritis in murine models (142). SNERV1/2 bind to the gp70-associated loci, *Sgp3*, and recruit KAP1 to repress transcription. SNERV deletions in New Zealand Black mice resulted in elevated NEERV transcripts and gp70 expression. These results indicate that defects in HERV repression may promote human lupus pathogenesis (127).

### Autism Spectrum Disorders

Activity of LINE-1 elements have additionally been implicated in many Autism Spectrum Disorders (ASD) phenotypes. Researchers have found a reduction of methylation and an increase in LINE-1 expression in ASD post-mortem brains (143, 144). ASD is a developmental disorder that impairs communication and behavior; however, little is known about the etiology of the disease. At the cellular level, researchers have found that individuals with ASD frequently show widespread inflammation and elevated brain cytokine expression. Additionally, a growing body of evidence supports the view that a chronic inflammation may contribute to autism symptomatology, with active neuroinflammatory processes being found throughout the brain in both cerebral cortex and cerebellum of patients with autism (145–148). Although new LINE-1 insertions seem to occur frequently in neurons, little is known about the contribution of this element in glial cells. It is becoming increasingly evident that under pathological conditions, there is a non-cell autonomous effect in the CNS, in which glial cells are as vulnerable as neurons (149). Astrocytes are often indicated as the contributors to disease phenotypes and in some instances, the disease initiators (150–152). Accumulating evidence links the cytokine dysregulation and persistent inflammatory phenotypes seen in mice and iPSC-derived models with astrocyte functional abnormalities [Figure 5; (153, 154)]. Indeed, ASD-derived astrocytes secrete elevated cytokines such as interleukin-6 (IL-6), which may interfere with proper neuronal development (155).

### Rett Syndrome

Rett Syndrome (RTT), once considered part of ASD, is an X-linked progressive neurodevelopmental disorder with autistic features, characterized predominantly by various mutations in the methyl CpG binding protein-2 (MeCP2) (156). Post-mortem brain tissue samples analyzed showed higher genomic LINE-1 ORF2 sequences in RTT patients when compared to controls (157). Muotri et al. demonstrated that MeCP2 loss of function increases susceptibility for LINE-1 insertions because the 5′UTR sequence within the LINE-1 promoter are targets of MeCP2-mediated transcriptional repression [Figure 2; (157)]. Conditioned media taken from RTT mutant astrocytes had adverse effects on wild type mouse neurons. After just 24 h, neurons grown in RTT astrocyte conditioned media had significantly smaller soma sizes, shorter neurites and less terminal ends (158). Re-expression of MeCP2 specifically in astrocytes improved locomotion, anxiety levels, and prolonged the lifespan of globally deficient MeCP2 mice. Even more, restoration of MeCP2 in astrocytes restored VGlut1 levels and dendritic morphology of neurons *in vivo* (159). More research must be conducted to confirm whether the abnormally high presence of LINE-1 retroelements seen in RTT contributes to: (1) the inflammatory response seen in astrocytes and (2) disease progression.

### Aicardi-Goutières Syndrome

Indeed, it has been found that the accumulation of LINE-1 copies in neurodevelopmental diseases promote inflammatory effects in astrocytes, as is in the case of AGS (38). AGS is a progressive inflammatory disorder that affects newborns and results in severe mental and physical handicap as well as greatly reduced lifespan. AGS can arise from mutations in three-prime repair exonuclease 1 (TREX1). Mutations in TREX1—which functions to degrade dsDNA/ssDNA—result in significant cytoplasmic accumulation of DNA species, which are then sensed as viral or “non-self.” This leads innate immune responses, such as the induction of IFN [Figure 5; (160)]. Thomas et al. demonstrated that a majority of those DNA species consist of cytoplasmic LINE-1 ssDNA. TREX-1 deficient NPCs expressed 70% more LINE-1Hs elements (38). Chronic RTi treatment of TREX1 deficient cell lines was shown to reduce cytoplasmic ssDNA to near control levels as well as improve neurite growth and decrease IFN secretion from astrocytes (38). In a 2018 phase II clinical trial for AGS patients, combinations of RTis were administered for 12 months to observe changes in interferon signaling (161). Across all patients who completed the study, the median interferon score dropped from 9.66 to 5.33. Interferon levels in serum and plasma were also reduced. Strikingly, global interferon-stimulated gene expression (ISG) decreased after 12 months of treatment but then returned to pre-treatment levels 6 months after discontinuing RTi treatment (161). Thus, RTi treatment can prove to be a promising therapeutic alternative for pathologies in which inflammation is a common denominator.

## Conclusion

Reviewed here are recent reports which highlight aberrant TE activation as contributors to a variety of neurological, neurodegenerative, and autoimmune pathologies. Activation of retroelements confer genomic and cellular instability as TEs can disrupt coding regions, rewire transcriptional networks, and modify epigenetic and post-transcriptional regulation of gene expression. Indeed, retrotransposons have evaded evolutionary attempts at repression and contribute to somatic mosaicism. In the diseased state, where repression or regulation of retrotransposons is diminished, expression of endogenous nucleic acids are upregulated, promoting a response from the host, similar to the one that occurs upon a viral infection or to environmental triggers. In most cases, the cell will initiate an interferon response. Persistent inflammation leads to functional abnormalities and disease phenotypes; therefore, we speculate that retroelement misregulation impacts human pathogenesis to a larger extent than previously thought.

## Author Contributions

AS wrote the manuscript with help from AM and ARM.

### Conflict of Interest Statement

ARM is a co-founder and has equity interest in TISMOO, a company dedicated to genetic analysis and brain organoid modeling focusing on therapeutic applications customized for autism spectrum disorder and other neurological disorders with genetic origins. The terms of this arrangement have been reviewed and approved by the University of California San Diego in accordance with its conflict of interest policies. The remaining authors declare that the research was conducted in the absence of any commercial or financial relationships that could be construed as a potential conflict of interest.
